# Extraction of a Large Stone from the Urethra of a Boy Three Years Old

**Published:** 1867-11

**Authors:** J. F. M. Davis

**Affiliations:** Gum Springs, Va.


### ARTICLE III.

*Extraction of a Large Stone from the Urethra of a Boy three years old.* By J. F. M. Davis, of Gum Springs, Va.

On the 1st of November, 1866, I was called to see a boy three years old, who the father told me had been suffering from gravel, and had not passed water for twenty-four hours preceding my arrival. I found my patient large for his age and apparently in good health, but was informed, however, that the child had been sick at intervals for a year or more.

I found the bladder greatly distended, and the skin over abdomen red. Not suspecting the cause of the retention of the urine, I ordered a warm bath, hoping in this way to relieve my little patient, but without effect. Learning that his bowels were constipated, I ordered an enema of warm soap-suds and common salt, which produced the discharge of hard lumps of fecal matter, without any discharge from the bladder. Not having a Catheter sufficiently small to introduce into the bladder of a child so young, I concluded to use an injection *per Urethra* of warm water, hoping in this way to overcome the difficulty; nothing, however, was accomplished.

Night coming on, I ordered anodynes and warm applications to be continued during the night.

I saw him again on the morning of the 2nd. He had rested tolerably well during the night, but his bladder was still unrelieved and greatly distended. I had supplied myself with a Catheter of the proper size and at once proceeded to introduce it into the bladder. About one inch from the meatus the catheter was arrested by the presence of a foreign body which from the rough and gritty feel was readily recognized as a stone of sufficient size to completely obstruct the urethra.

After a careful examination it was made evident that there was no means of removing the obstruction except by an operation.

After forcibly drawing back the prepuse an incision was made through it to the urethra. Firm pressure was now made behind the stone with the hope of forcing it sufficiently near the mouth of the urethra to grasp it with a pair of forceps; but it was found impossible to dislodge it. The incision was now extended through the urethra and a stone half an inch long and three-quarters of an inch in circumference was removed, to the great relief of the little sufferer. There was an immediate and copious flow of urine. The hemorrhage was slight, and by the application of cold water soon ceased. The after treatment consisted in the application of cold water by means of cloths. For some days after the operation small clots of blood passed at each micturition.—Nothing unpleasant occurred, and the wound healed in a few days.

Ten days after the operation I was called to see my little patient again, and found him extremely ill with acute meningitis. It proved a tedious attack, but he finally recovered and now seems in the most perfect health.

